# Association of ABO Blood Group and Body Mass Index: A Cross-Sectional Study from a Ghanaian Population

**DOI:** 10.1155/2018/8050152

**Published:** 2018-03-27

**Authors:** Samuel Smith, Isaac Okai, Chrissie Stansie Abaidoo, Emmanuel Acheampong

**Affiliations:** ^1^Department of Medical Laboratory Technology, Kwame Nkrumah University of Science and Technology (KNUST), Kumasi, Ghana; ^2^Department of Anatomy, School of Medical Sciences, Kwame Nkrumah University of Science and Technology (KNUST), Kumasi, Ghana; ^3^Department of Molecular Medicine, School of Medical Sciences, Kwame Nkrumah University of Science and Technology (KNUST), Kumasi, Ghana

## Abstract

ABO blood group and body mass index (BMI) have individually been appraised as risk factors for certain diseases. From statistical perspective, it may be important to examine the relationship between the ABO blood antigen and BMI. This cross-sectional study involved 412 participants aged 18 to 46 at the Kwame Nkrumah University of Science and Technology (KNUST), Kumasi. Weight and height of participants were measured for BMI calculation; blood group determination was done using antisera. Blood group O was the most prevalent (51.2%), while Rhesus-positive individuals constituted 90.3%. 6.3% of the participants were obese, while 18.7% were overweight. There was significant (*p*=0.006) higher prevalence of obesity in females (10.3%) than in males (3.4%). The study did not observe any significant difference by association of ABO blood group with gender (*p*=0.973), BMI (*p*=0.307), or Rhesus status (*p*=0.723). Regarding gender (*p*=0.400) and BMI (*p*=0.197), no statistically significant difference was observed between Rhesus blood groups. The prevalence of overweight, obesity, blood type O, and rhesus positive observed among students in this study is largely similar to what has been reported in published studies in Ghana and from other countries. Overweight and obesity were not associated with ABO blood groups or Rhesus in this study.

## 1. Introduction

The distribution pattern of the ABO blood antigen varies by the prevalence type among different populations in the world. The association between ABO blood group and several elements of the human population such as intelligence [1], socioeconomic status [2], diet [3], diseases [4], and others has long been suggested. Some of these reports such as from Gibson et al. [1] and Beardmore and Karimi-Booshehri [2] linking ABO blood type to intelligence and socioeconomic class, respectively, have been decades old, and the potential mechanism by which ABO antigens determine these consequences was not underscored. Furthermore, D'Adamo's [3] popular blood type diet, without proven scientific evidence, was theoretically based on the belief that each ABO blood group carries the genetic information of their diets [5]. Thus, Cusack et al. [5] in a systematic review of published data on blood type diet concluded that there was no scientific proof to assess the effectiveness of the blood type diet. The authors recommended a validation study for the purported health benefits of blood type diets. From this premise, Wang et al. [6] conducted the first study to examine the association between blood type diets and biomarkers of cardiometabolic health in experimental and control groups. They concluded that some benefits that may be derived from adherence to the one's blood type diet are not directly attributable to the individual's ABO blood status; therefore, blood type diet hypothesis is not valid scientifically.

Nonetheless, the relationship between certain diseases and ABO blood type probably appears to have received good attention over the past five decades. Some reports have shown evidence of potential association between ABO blood and some diseases. However, it has yet to be fully established the validity or otherwise of how the statistical associations between blood group and some disease risks actually translate in vivo; evidence suggests that blood group antigens may play a biological role in some disease pathogenesis [7]. ABO antigen type has been postulated from many reports as a risk factor for some cardiovascular diseases [8–10], cancers [11–14], and infectious diseases [15–17]. The proposed mechanism believed to underlie how ABO blood group may influence cardiovascular disease risk involves the possible regulatory effect of ABO antigens on plasma levels of von Willebrand factor (VWF) and coagulation factor VIII (FVIII) [18, 19]. This mechanism is corroborated by the observation that individuals with non-O blood group have circulating levels of both VWF and FVIII that are approximately 25% higher than those in O blood group subjects [18]; the presence of ABH antigenic structures on circulating VWF modulates their activity through specific glycosyltransferase enzymes [20]. Considering malignancies, the exact mechanism by which the ABO blood antigen may function in cancer pathogenesis is generally not known; however, one theory suggests that circulating levels of several proinflammatory and adhesion molecules which play a key role in the tumorigenesis process may be regulated by ABO blood antigens [21].

Body mass index (BMI), a measure of excess body weight, is useful for assessing aspects of health in children and adults within a population. Based on the WHO classification of BMI, an individual may be clinically considered obese, overweight, normal, or underweight. BMI pattern of distribution differs within and between different populations globally; changing trends in BMI of individual populations are known and linked to changes in socioeconomic status [22, 23]. Obesity and overweight are known to be harmful to health [24], and many studies have demonstrated the association of increased BMI and risk of development of certain diseases. Excess body weight is believed to accentuate the risk of numerous diseases and clinical disorders, such as coronary heart disease, strokes, cancers, type 2 diabetes mellitus, hypertension, asthma, liver disease, psychopathological conditions [25], and allergic diseases [26].

Although ABO blood group and BMI have individually been appraised as risk factors for certain illnesses, few studies [27–40] have been conducted to examine whether carrying a particular ABO blood antigen potentially predisposes one to higher body mass index. These studies, however, have arrived at different conclusions on whether ABO status associates or does not associate with BMI. Due to the racial and ethnic disparities existing among different people globally, population-based studies are relevant. To our knowledge, no study has been done to test the association of BMI and ABO blood group in a Ghanaian sample. Therefore, this study was undertaken to examine the distribution and association of these two risk factors in a Ghanaian setting.

## 2. Methodology

### 2.1. Study Design and Setting

This cross-sectional study was conducted in KNUST from April 2017 to May 2017 among undergraduate students. KNUST is a public university located in Kumasi in the Ashanti Region of Ghana. The main university campus, which is about 7 square miles in area, is situated about 8 miles (13 km) to the east of Kumasi. There are 6 halls of residence at the main campus. The university has about 40,000 undergraduate students and consists of 6 colleges.

### 2.2. Study Population and Subject Selection

Using a simple random sampling stratified by the 6 colleges, a total of 412 students from the first to the fourth academic years were recruited for the study which included 238 males and 174 females of ages 18–46 years. Individuals with physical deformities that influenced height such as short or amputated lower limbs, kyphosis, and scoliosis, as well as those with bleeding disorders, were excluded from the study.

### 2.3. Sample Size Determination

A total of 412 participants were recruited from a population of 40,000 students at KNUST using an assumed distribution response rate among the respondents at 50%, at 95% confidence interval (*z*-score 1.96). Using the Cochran formula [41], the minimum size required was 381; however, to accommodate a nonresponse rate of 10.0% and stronger statistical power and effect size, the sample size was projected to 412 students.

### 2.4. Anthropometric Measurement and ABO Blood Group Analysis

Weight of the students was measured in the upright position to the nearest 0.5 kg using a weight measuring scale (Seca, Hamburg, Deutschland). Height was measured without shoes to the nearest 0.1 cm with a Shahe stature meter (Shanghai, China). BMI was calculated based as weight in kilograms divided by the square of the height in metres (kg/m^2^). Blood samples were collected for ABO blood group analysis.

### 2.5. Categorization of BMI

BMI was classified according to the proposed criteria of the WHO [18], where BMI of the following values: <18.5 kg/m^2^, 18.5–24.9 kg/m^2^, 25–29.9 kg/m^2^, and ≥30 kg/m^2^, is categorized as underweight, normal weight, overweight, and obese, respectively.

### 2.6. Ethical Consideration

Ethical approval (Ref.: CHRPE/AP/231/17) for the study was obtained from the Committee on Human Research, Publication and Ethics (CHRPE) of the School of Medical Sciences (SMS), Kwame Nkrumah University of Science and Technology (KNUST). Participation was voluntary, and written informed consent was obtained from each participant. Respondents were assured that the information gathered was to be used strictly for research and academic purposes only. In addition, respondents were given the freedom to opt out any time they thought they could not continue with the study.

### 2.7. Statistical Analysis

SPSS version 20.0 statistical software package was used to carry out statistical analysis. Descriptive statistics of the mean standard deviation and standard error was used to examine the data. Student's *t*-test for nonparametric data was used to compare the difference between the means of the two investigated parameters. The Pearson chi-square correlation analysis was used to determine the association between BMI and ABO blood group. Percentages for independent variables were calculated, and *p* < 0.05 was considered statistically significant.

## 3. Results and Discussion

As shown in Table 1, of 412 students, 57.5% of the participants were male and 42.5% were female. The mean age was 23 years with the majority (70.0%) of participants between 21 and 25 years. Blood group O was the most prevalent (51.2%), followed by B (26.0%), A (19.7%), and AB (3.1%). 90.3% of individuals were Rhesus-positive, while 9.7% were Rhesus-negative. Also, 6.3% (26/412) of the participants were obese, while 18.7% (77/412) were overweight (Table 1).

Obesity was significantly (*p*=0.006) prevalent among females (10.3%; 18/175) than males (3.4%; 8/237). The study did not observe any significant difference regarding the ABO blood group in relation to gender, BMI, and Rhesus blood group (Table 2). Moreover, Rhesus status of participants was not significantly associated with either BMI or gender (Table 3). The prevalence of obesity and overweight observed in this study is similar to that of a cross-sectional study conducted among sampled students in Kumasi, Ghana, by Kumah et al. [42] but higher than that of Obirikorang et al.'s study [43]. Studies on overweight and obesity rates among students from other countries have demonstrated a wide variety of prevalence. For example, obesity and overweight rates were 10.4% among girls and 3.2% among boys in Uganda and Ghana [44]. In Malaysia [45] and Pakistan [29], the rate among students was 14.8% and 16.0%, respectively. However, studies carried out among public workers in Ghana had contrasting results [46]. It is possible that geographical and sample size differences may underlie the disparities in prevalence among the various reports. The high prevalence of overweight and obesity observed in females from our study is analogous to reports from studies by Kumah et al. [42] and Obirikorang et al. [43] in Ghana, Armstrong et al. [47] in South Africa, and Hamaideh et al. [48] in Jordan. In our cultural setting, overweight and obesity are seen as a sign of affluence and prosperity. As a result, most African women aspire to an increased BMI so as to be accepted. This cultural ideology is contrary to those of the western parts of the world who view females with a lower BMI to be more attractive. Additionally, males are naturally more active and tend to expend energy reserves as compared to females.

Blood type O was the prevalent ABO blood group in the present study. This trend of result is similar to the report from studies by Acquaye [49] in Ghana and Eru et al. [50] in Nigeria. Also, studies by Bhatti et al. [51] in India and Parveen et al. [30] as well as Bhattacharyya et al. [52] in Pakistan have reported similar ABO blood group pattern. Worldwide distribution pattern has shown blood type O to be the most prevalent blood group followed by group B, group A, and group AB [53], which is consistent with the findings in this present study. Nonetheless, a study done among a Turkish population found blood group A to be the most widely distributed [54]. Blood group O is hypothesized to offer the maximum protection to people who live in areas endemic for infectious diseases. Hence, the incidence of this blood group is very high in tropical regions of the world where infectious diseases are common [55]. The clinical importance of this distribution is illuminated by the low malaria parasitemia seen in individuals with the blood group O who live in West Africa [15].

Reports in literature on the relationship between ABO blood group and BMI are inconsistent [38, 56], with various authors associating increased BMI with the presence of particular ABO antigens, while others have shown no association between these two factors. Significant association was seen between ABO blood group and BMI among sampled populations from Pakistan [30], India [33, 34, 38], Malaysia [31, 32], Nigeria [35], and Denmark [40]. One may be tempted to assume that ABO blood type and body weight may be biologically related probably through a pathway that involves thrombotic factors like FVIII because it is known for example, that non-blood group O individuals have higher FVIII. For instance, increased BMI was associated with a higher level of FVIII [57, 58]. Also, obesity is considered to be an inflammatory disease [58], and this phenomenon may be linked to a hypothesized ABO blood antigen regulatory effect on inflammation [21]. Nevertheless, such a molecular pathway or any others have not been found, suggesting that association between ABO and BMI may be arbitrary.

The present study did not observe any significant association between ABO blood group and BMI. Our observation agrees with findings from large cross-sectional studies by Jafari et al. [28] among different ethnicities in Pakistan and other studies by Aboel-Fetoh et al. [27] in Saudi Arabia, Ainee et al. [39] in Sargodha District, Chuemere et al. [36] in Nigeria, and Mascie-Taylor and Lasker in the UK [37]. The absence of any significant association between ABO and BMI in our study may not be surprising as even larger cohort studies like those by Jafari et al. [28] and Mascie-Taylor and Lasker [37] failed to link BMI with either ABO or Rh phenotype. Furthermore, to our knowledge, it is not established from any genome-wide association studies in literature whether some genes such as *FTO, LEP, LEPR, MC4R, NPY2R,* and *POMC* that have been implicated in higher BMI are located on the same chromosomal region as that of ABO or that ABO gene exerts regulatory control over them. It is therefore plausible to say that a genetic basis for linking ABO to BMI is sufficiently lacking. In our opinion, the different observations made regarding the relationship between ABO/Rh status and BMI may be practically dependent on local factors that modify population phenotype [37] or sample size variation rather than actual genetic influence. Collectively, our findings are comparable to reports from other studies [27, 28, 39, 42–51]. However, being an institutional-based cross-sectional study, we recognize that making generalization of our findings to the Ghanaian population may be inappropriate.

## 4. Conclusion

The prevalence of overweight, obesity, blood type O, and Rhesus positive observed among students in this study is largely similar to what has been generally reported in literature. However, overweight and obesity were not associated with ABO blood groups or Rhesus in this study contrary to the view of some reports. There is the need for educational-based multiple strategies to combat the increasing rate of obesity and overweight among students.

## Figures and Tables

**Table 1:** Descriptive statistics of study participants (*n*=412).

Variables	Mean ± SD	Percentage
Age (years)	23.0 ± 4.0	
Height (cm)	169.8 ± 8.6	
Weight (kg)	64.9 ± 13.1	
BMI (kg/m^2^)	22.5 ± 4.0	
*Age groups (years)*	*Frequency*	
≤20	19	19.2
21–25	280	70.0
26–30	79	7.5
>30	31	5.3
*Gender*		
Male	237	57.5
Female	175	42.5
*ABO blood groups*		
A	81	19.7
AB	13	3.1
B	107	26.0
O	211	51.2
*Rhesus (−/+)*		
Negative	40	9.7
Positive	372	90.3
*BMI categories*		
Underweight	49	11.9
Healthy	260	63.1
Overweight	77	18.7
Obese	26	6.3

SD  standard deviation *n*  number of participants.

**Table 2:** Association between ABO blood group and BMI, Rh blood group, and gender of the participants.

Variables		ABO blood group, *n* (%)			*X* ^2^, df	*p* value
	A (*n*=81)	AB (*n*=13)	B (*n*=107)	O (*n*=211)		
*BMI*						
Underweight	11 (13.6%)	3 (23.1%)	15 (14.0%)	20 (9.5%)	10.56, 9	0.307
Normal	50 (61.7%)	8 (61.5%)	62 (57.9%)	140 (66.4%)		
Overweight	14 (17.3%)	0 (0%)	21 (19.6%)	42 (19.9%)		
Obese	6 (7.4%)	2 (15.4%)	9 (8.4%)	9 (4.3%)		
						
*BMI (mean ± SD)*	22.5 ± 4.2	22.4 ± 5.2	22.3 ± 4.1	22.5 ± 3.8		0.964 (ANOVA)
*Rh blood group*					1.32, 3	0.723
Positive	71 (87.7%)	12 (92.3%)	99 (92.5%)	190 (90.0%)		
Negative	10 (12.3%)	1 (7.7%)	8 (7.5%)	21 (10.0%)		
*Gender*					0.23, 3	0.973
Male	45 (55.6%)	8 (61.5%)	62 (57.9%)	122 (57.8%)		
Female	36 (44.4%)	5 (38.5%)	45 (42.1%)	89 (42.2%)		

SD: standard deviation; *n*: number of participants; *X*^2^: chi-square; df: degree of freedom; BMI: body mass index; ANOVA: analysis of variance; *p* < 0.05 is statistically significant.

**Table 3:** Association between Rh blood group and BMI and gender of the participants.

Variables	Rhesus blood group, *n* (%)		*X* ^2^, df	*p* value
	Negative (*n*=40)	Positive (*n*=372)		
*Gender* ^a^				0.400
Male	26 (65.0%)	211 (56.7%)		
Female	14 (35.0%)	161 (43.3%)		
*BMI (kg/m* ^*2*^)^b^			4.68, 3	0.197
Underweight	4 (10.0%)	45 (12.1%)		
Normal	29 (72.5%)	231 (62.1%)		
Overweight	3 (7.5%)	74 (19.9%)		
Obese	4 (10.0%)	22 (5.9%)		

BMI: body mass index; *n*: number of participants; *X*^2^: chi-square; df: degree of freedom;^a^Fisher's exact test; ^b^chi-square test; *p* < 0.05 is statistically significant.
